# The Predictive Value of ACR TI-RADS Classification for Central Lymph Node Metastasis of Papillary Thyroid Carcinoma: A Retrospective Study

**DOI:** 10.1155/2022/4412725

**Published:** 2022-02-07

**Authors:** Minying Zhong, Zhaoming Zhang, Yisheng Xiao, Yanping He, Yongyu Chen, WeiJun Huang, Liping Lu

**Affiliations:** ^1^Department of Ultrasound, The First People's Hospital of Foshan, Foshan 528000, China; ^2^Department of Orthopedics, Affiliated Foshan Hospital, Guangzhou University of Traditional Chinese Medicine, Foshan 528000, China; ^3^The First Clinical Medical College of Guangzhou University of Traditional Chinese Medicine, Guangzhou 510000, China; ^4^Department of Ultrasound, The Sixth Affiliated Hospital of South China University of Technology, Foshan Nanhai District People's Hospital, Foshan 528000, China

## Abstract

**Background:**

The aim of this retrospective study was to evaluate the risk factors for central lymph node metastasis (CLNM) in papillary thyroid carcinoma (PTC), according to the guidelines of the 2017 Thyroid Imaging Report and Data System (TI-RADS) published by the American College of Radiology (ACR).

**Methods:**

This study included a retrospective analysis of 844 patients with PTC who were pathologically diagnosed, treated with central lymph node dissection, and divided into CLNM and nonmetastatic groups. Univariate and multivariate analyses were performed to determine the relationship between the TI-RADS score and CLNM.

**Results:**

Among 844 patients, 439 developed CLNM, with a metastasis rate of 52% and a TI-RADS score of 9.42 ± 2.262, which were higher than those of the non-CLNM group (*P* < 0.05). Univariate analysis demonstrated that the sex, location, maximum diameter of the nodule, multifocality, margin, shape, calcification, and TI-RADS score were related to CLNM (*P* < 0.05 for all). However, multivariate logistic regression analysis demonstrated that female, maximum diameter of the nodule, multifocality, a taller-than-wide shape, and high TI-RADS score were the independent risk factors for CLNM (*P* < 0.05 for all).

**Conclusion:**

The TI-RADS score combined with sex, nodule size, shape, and multifocality has a certain predictive effect on CLNM, which can provide a reference to the clinicians for further treatment strategies.

## 1. Introduction

Thyroid cancer (TC) is one of the most common endocrine tumors in the head and neck. In recent years, the incidence rate of TC has increased year by year. Papillary thyroid carcinoma (PTC) is the most common pathological type, which accounts for 80% [1] of all thyroid malignancies. Central lymph node metastasis (CLNM) is the most common route of PTC with a metastasis rate of 20% to 90% [2, 3]. The classification standard developed by the American College of Radiology (ACR) Thyroid Imaging Reporting and Data System (TI-RADS) in 2017 has a high clinical application value [4] and helps to standardize the management of thyroid nodules in China. However, there are few reports on the use of TI-RADS to predict CLNM. Therefore, this study mainly explored the risk factors of CLNM in patients with PTC and its risk relationship with TI-RADS to guide clinical decision making.

## 2. Methods

### 2.1. General Information

A total of 844 patients with PTC, who were surgically treated and diagnosed by histopathology at the First People's Hospital of Foshan and People's Hospital of Nanhai District, Foshan, between January 2016 and February 2021, were enrolled. They consisted of 268 (31.8%) males and 576 (68.2%) females aged from 11 to 84 years with an average of 41.98 ± 12.77 years. The maximum diameter of the nodules was 16.43 ± 11.07 mm (range: 3–94 mm). This study was approved by the First People's Hospital of Foshan and People's Hospital of Nanhai district, Foshan. An informed consent form was signed by all the patients, and patients who had relative contraindications were excluded. Inclusion criteria were as follows: (i) thyroid surgery was performed for the first time, and the postoperative pathology was PTC, (ii) central lymph node dissection was performed, and (iii) there was no history of other malignant tumors.

### 2.2. Data Collection

Ultrasonic examinations were performed with Yum MyLab Class C and GE Logiq 9 sonographic scanners equipped with high-frequency, 5e12-MHz linear probes. Data were collected about the sex and age of the patients and size and location of the thyroid nodules. The ultrasound images were analyzed retrospectively by two doctors with more than 5 years of experience in thyroid diagnosis.

According to the TI-RADS classification, the following ultrasound features of the nodule were recorded: composition, echo, aspect ratio, margin, and calcification. Scores were assigned according to the degree of each ultrasound feature. The final scores were added to calculate the total TI-RADS score, and in case of disagreements, the results were approved after discussion.

### 2.3. Statistical Methods

Statistical analyses were performed using SPSS version 22.0. Mean ± standard deviation was used to express normal distribution of measurement data (age, maximum diameter, and TI-RADS score). Two independent-sample *t*-tests were performed for measurement data with normal distribution. Count data (sex, location, and ultrasound characteristics) are described as percentages. The chi-squared test was performed to analyze the count data of the hypothesis test. The risk factors for lymph node metastasis in the central area were identified, and variables that were significant for univariate analysis were included in the multivariate logistic regression analysis. *P* values of <0.05 indicated that the difference was statistically significant.

## 3. Results

### 3.1. General Information

844 patients with PTC were divided into the CLNM and nonmetastasis group by postoperative histopathology. 439 patients in the CLNM group had a metastasis rate of 52% (439/844) and consisted of 166 (37.8%) males and 273 (62.2%) females. The mean TI-RADS score was 9.42 ± 2.262, which was higher than that in the group without CLNM (*P* < 0.001) (Table 1).

### 3.2. Single-Factor Analysis of Lymph Node Metastasis in the Central Area of PTC

Sex, location, multifocality, shape, margin, calcification, maximum diameter of the nodule, TI-RADS total score, and CLNM had a significant relationship (*P* < 0.001), but there was no statistical difference in the relationship between age, composition, echo, and CLNM (*P* > 0.05) (Table 2).

### 3.3. Multivariate Analysis of Lymph Node Metastasis in the Central Area of PTC

Eight factors with statistical significance in the univariate analysis were included in the multivariate logistic regression analysis. The results showed that the larger the nodule diameter and the higher the TI-RADS score, the higher the risk of CLNM, which was statistically significant (OR = 1.022, 95% CI 1.006–1.038, *P*=0.006; OR = 1.359, 95% CI 1.219–1.516, *P* < 0.01); female, taller-than-wide shape, and multifocal increased the risk of CLNM, and the difference was statistically significant (OR = 1.839, 95% CI 1.313–2.575, *P* < 0.01; OR = 4.443, 95% CI 2.771–7.125, *P*=0.01; and OR = 1.660, 95% CI 1.200–2.296, *P* < 0.01) (Table 3).

## 4. Discussion

The central area of the lymph nodes is the first area of PTC metastasis. It has been reported that the metastasis rate is 20%–90% [2, 3, 5]. Currently, it remains controversial whether patients with node-negative (cN0) PTC should undergo preventive dissection of the central area lymph nodes or not. When the American Thyroid Association (ATA) guidelines [6, 7] were updated from the 2009 version to the 2015 version, there were reservations about preventive central region lymph node dissection. The ATA [7] indicated that if CLNM is not clear, it is not recommended to perform lymph node dissection, since the surgery may easily lead to postoperative complications such as recurrent laryngeal nerve injury. Additionally, prophylactic lateral lymph node dissection is not recommended owning to the significant risks and lack of impact on survival. However, some scholars [8] believed that the detection rate and sensitivity of ultrasound to CLNM are low, so some guidelines [9] recommend preventive central lymph node dissection in consonance with the current condition in China.

The metastasis rate of CLNM in this study was 52%, which was consistent with the previously reported metastasis rate of 38%–60.9% [10–12]. The univariate analysis of this study demonstrated that the risk factors associated with CLNM included the sex, location, multifocality, shape, margin, calcification, large diameter of the nodule, and high TI-RADS score. Multivariate regression analysis found that five items, including female, maximum diameter of the nodule, a taller-than-wide shape, high TI-RADS score, and multifocality, were the independent risk factors for CLNM.

The incidence rate of PTC in females is higher than that in males, but males are more likely to develop CLNM [13]. A possible explanation for this could be that females are affected by estrogen and progesterone while males' basal metabolism is strong and tumor proliferation is active.

This study included more females (62.2%) than males (37.8%) in CLNM (*P* < 0.001). Compared with males, females increased the risk of CLNM (OR = 1.839, 95% CI 1.313–2.575, *P*=0.01), which may be related to the large sample number of females in this study and was consistent with previous studies [14]. Currently, information on the relationship between the tumor location and CLNM is not settled. Some studies have reported [15] that tumors occupying the entire lobe and located in the upper and middle lobes are risk factors for CLNM. Nonetheless, some researchers believe that the location of tumors in the middle and lower lobes is a risk factor for CLNM [16]. In this study, we observed that tumors located in the upper lobe increased the possibility of CLNM, and this result was consistent with that of relevant studies [15].

A considerable number of studies have confirmed that CLNM is closely related to the size of nodules and the risk of metastasis increases with the increase in its size [17–19]. Additionally, we observed that the maximum diameter of the nodule in the CLNM group (18.42 ± 12.057) was significantly larger than that in the non-CLNM group (14.28 ± 9.458). The multivariate analysis demonstrated that the risk of CLNM increased with every 1 mm increase in the maximum diameter of the nodule. The nodules with taller-than-wide shape were related to the malignancy of thyroid nodules [20]. This study found that nodules with taller-than-wide shape were more likely to develop central lymph node metastasis than those without it, which was similar to the results reported in other study [21]. But, its reliability still needs to be further verified. The related reports have presented that CLNM of multiple tumors is higher than that of single tumors [22]. Our study also observed that the multifocality was an independent risk factor. This was consistent with TANG T's opinion [23], which stated that the more the tumor lesions, the greater the occurrence of CLNM, when tumor with more than three lesions is an independent risk factor of CLNM.

Our study observed that extranodal invasion was 51.7% (227/439) and irregular/lobular shape was 24.4% (107/439) in the CLNM group. Different manifestations of nodular margins have a statistically significant effect on CLNM, which is consistent with relevant studies [18, 24]. When the tumor protrudes from the envelope, it is more aggressive and more likely to cause bloody or lymph node metastasis. Microcalcification in a nodule is an important aspect of distinguishing between benign and malignant nodules. It has high specificity and is pathologically manifested as grit. It is closely related to multifocal, aggressive, and lymph node metastases of the nodule [25, 26]. Our study found that the microcalcification was 70.2% (308/439) in the CLNM group, and previous reports also indicated that [26] intranodular microcalcification was statistically significant in predicting PTC cervical lymph node metastasis, which was consistent with the results of our study.

**Figure 1:**
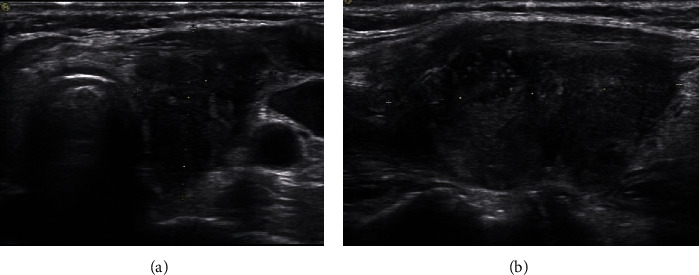
Papillary thyroid carcinoma with central lymph node metastasis. The tumor is located in the middle and lower poles of the left thyroid lobe. The maximum diameter is 40 mm. It is solid and very hypoechoic with an aspect ratio <1, external invasion, internal visible hyperechoic small calcification, and American College of Radiology Thyroid Imaging Reporting and Data System (ACT TI-RADS) score of 11 points. (a) Transverse section and (b) longitudinal section.

**Figure 2:**
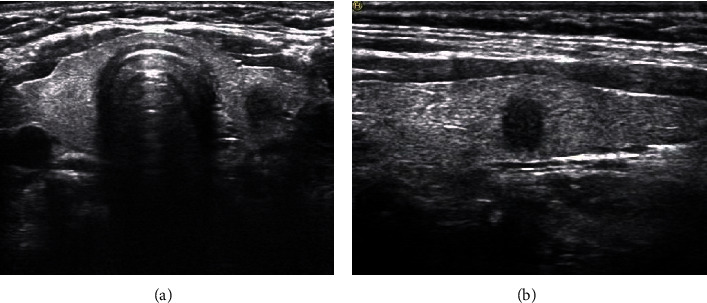
Ultrasonography of the nodules in papillary thyroid carcinoma (PTC) without central lymph node metastasis. The tumor is located in the middle of the left thyroid lobe, with a maximum diameter of 8 mm, solid and very hypoechoic, aspect ratio >1, and unclear margin. The American College of Radiology Thyroid Imaging Reporting and Data System (ACT TI-RADS) score was 8 points. (a) Transverse section; (b) longitudinal section.

The central lymph nodes are located between the hyoid bone and sternum, over the trachea close to the thyroid. Owing to their deep location, the sensitivity and specificity of ultrasound for the diagnosis of CLNM are low [27]. Previous studies have shown that, among the current five international guidelines for the management of thyroid nodules, 2017 ACR-TI-RADS showed the highest specificity and has the highest correlation between sensitivity and specificity in identifying malignant lesions [28]. This study indicated that the TI-RADS score of the CLNM group (9.42 ± 2.262) (Figure 1) was significantly higher than that of the non-CLNM group (8.32 ± 2.819) (Figure 2). The multivariate analysis also demonstrated that, for every increase by one point in the TI-RADS score, the risk of CLNM increased. This suggested that attention must be paid to the risk of lymph node metastasis in the central area for nodules with high scores.

This study had some limitations. Since it was a retrospective analysis, there were some confounding factors. Scoring and classification based on static two-dimensional images of the nodules reduced the accuracy to a certain extent, leading to possible deviations in the results.

## 5. Conclusions

In summary, the results of this study indicated that female, maximum diameter of the nodule, multifocality, a taller-than-wide shape, and high TI-RADS score were the independent risk factors for CLNM. When scoring the nodule with TI-RADS during ultrasonic examination, we can predict whether there is CLNM or not in combination with size, shape, multifocality, etc., which can provide the clinicians with the reference basis for a surgical approach.

## Figures and Tables

**Table 1:** Single-factor analysis of lymph node metastasis in the central area of PTC.

Term	CLNM		*χ* ^2^/*t*	*P* value
	Positive (*n* = 439)	Negative (*n* = 405)		
Gender			15.502	<0.001
Male	166	102		
Female	273	303		

Age (years)			3.045	0.069
≤45	292	245		
>45	147	160		

Location			28.972	<0.001
Upper	51	96		
Middle	236	219		
Lower	242	90		

Mulifocality			19.322	<0.001
Solitary	247	287		
Multifocal	192	118		

Maximum diameter of the nodule	18.42 ± 12.057	14.28 ± 9.458	−5.572	<0.001
TI-RADS total score	9.42 ± 2.262	8.32 ± 2.819	−6.211	<0.001

CLNM: central lymph node metastasis.

**Table 2:** Single-factor analysis of lymph node metastasis in the central area of PTC.

Term	CLNM		*χ* ^2^/*t*	*P* value
	Positive (*n* = 439)	Negative (*n* = 405)		
Composition			1.09	0.58
Cystic	0	1		
Spongy nodules	0	0		
Mixed nodules	20	18		
Substantial or almost substantial	419	386		

Echo			4.431	0.219
No echo	1	0		
Highly echoic	26	18		
Low echo	323	370		
Very low echo	55	51		

Shape			15.25	<0.001
Wider than tall	294	218		
Taller than wide	145	187		

Margin			83.186	<0.001
Smooth	3	31		
Owed	102	170		
Irregular/lobed	107	98		
External invasion	227	106		

Calcification (multiple choice)			55.164	<0.001
Without or with comet tail sign	82	166		
Coarse	25	25		
Peripheral (circular)	9	11		
Punctate echo	308	191		
Coarse + surrounding	1	1		
Coarse + punctate strong echo	14	11		

**Table 3:** Multivariate analysis of lymph node metastasis in the central area of PTC.

		B	S. E	Wals	Sig.	Exp (B)	95% CI
Sex	Male	0.609	0.172	12.575	0.01	1.839	1.313–2.575
	Female						

Location	Upper			21.147	0.01		
	Middle	−1.115	0.243	21.100	0.01	0.328	0.204–0.528
	Lower	−0.396	0.184	4.606	0.032	0.673	0.469–0.966

Maximum diameter		0.022	0.008	7.643	0.006	1.022	1.006–1.038
Shape		1.491	0.241	38.307	0.01	4.443	2.771–7.125
Margin	Smooth			7.017	0.071		
	Owed	−1.555	0.679	5.250	0.022	0.211	0.056–0.799
	Irregular/lobed	−0.269	0.241	1.250	0.264	0.764	0.477–1.225
	External invasion	−0.341	0.200	2.912	0.088	0.711	0.480–1.052

TI-RADS score		0.307	0.056	30.543	0.01	1.359	1.219–1.516
Multifocality		0.507	0.165	9.373	0.002	1.660	1.200–2.296

## Data Availability

The data used to support the findings of this study are available from the corresponding author upon request.
